# Kinetics of extracting valuable components from Ti-bearing blast furnace slag by acidolysis with sulphuric acid

**DOI:** 10.3389/fchem.2024.1369937

**Published:** 2024-02-08

**Authors:** Yan Wang, Xin Gao, Siqi He, Jun Guo

**Affiliations:** ^1^ College of Environment and Resources, Southwest University of Science and Technology, Mianyang, Sichuan, China; ^2^ Central Station of Ecological Environmental Monitoring in Mianyang, Mianyang, Sichuan, China; ^3^ College of Resources and Environmental Engineering, Mianyang Teachers’ College, Mianyang, Sichuan, China

**Keywords:** Ti-bearing blast furnace slag, concentrated sulphuric acid, acidolysis, reaction process kinetics, sulphuric acid

## Abstract

Ti-bearing blast furnace slag is a kind of solid waste produced by Pangang Group Company through the blast furnace smelting method. A variety of valuable components can be extracted from the Ti-bearing blast furnace slag after acidolysis with concentrated sulphuric acid. In order to study the kinetics of acidolysis, this paper investigated the effects of the acidolysis temperature, acid-slag ratio and raw material particle size on the overall extraction rate of Ti^4+^, Mg^2+^ and Al^3+^ components at different reaction times, and simulated the acidolysis process by using the unreacted shrinking core model. The results showed that the acidolysis process was controlled by internal diffusion with an apparent activation energy of 19.05 kJ mol^–1^ and the semi-empirical kinetic equation of the acidolysis process was obtained.

## 1 Introduction

Vanadium-titanium magnetite in the West Panzhi region produces an industrial solid waste, Ti-bearing blast furnace slag (TBFS), in the process of ironmaking, and the mass fraction of TiO_2_ in the TBFS ranges from 18% to 22% (Zhang et al., 2007). Currently, the main disposal method for TBFS is to place it in a slag disposal pit, which has the disadvantages of requiring a large area, as well as the potential threat of environmental pollution and to human and animal health (Kuwahara et al., 2010). Therefore, it is essential to explore effective treatment methods for TBFS.

Due to the high titanium content in TBFS, it is difficult to use it directly for construction materials in the resource utilisation process (Huang et al., 2016; He et al., 2019). Therefore, researchers in China have focus on extracting valuable components from TBFS, which is considered to be more significant for research and economic value. As early as the “seventh Five Year Plan” and “eighth Five Year Plan” periods, Panzhihua Iron and Steel Research Institute conducted research on the extraction of titanium from TBFS. Currently, a production line for titanium extraction by chlorination with an annual treatment of 180,000 tons of TBFS has been established (Huang and Zhang, 1994). However, another industrial solid waste, titanium extraction tailings, is generated in the chlorination process (Peng et al., 2005). In addition, researchers have investigated the extraction of Ti, Si, Mg, Al and other elements from TBFS using alkalis, salts, acids, and other additives to prepare corresponding chemical products (Zhou et al., 1999; Zhou et al., 2013). Among the various extraction processes, sulphuric acidolysis has rapidly become a research hotspot because of its mature process technology, stable and easy-to-control reaction, simple operation and the ability to extract a variety of valuable components simultaneously. In the process of using sulfuric acid to hydrolyze TBFS, whether dilute or concentrated sulfuric acid as a reaction agent, or use one stage leaching (Jiang et al., 2010), or two stage leaching (Nie et al., 2023a), or roasting (He and Wang, 2023), or hydrothermal as reaction method, the extraction rate for titanium is high using this method (Valighazvini et al., 2013; Zhang et al., 2022; Li et al., 2024a). The acidolysis of TBFS using sulfuric acid can convert solid phase Ti, Mg, and Al into soluble Ti^4+^, Mg^2+^ and Al^3+^, which are then sequentially separated by boiling hydrolysis and stepwise precipitation (Hongjuan et al., 2015). Therefore, optimizing the acidolysis conditions, improving the extraction rate, and efficiently extracting the valuable components in TBFS are of great significance to achieve the dual purposes of waste treatment and resource utilisation.

At present, domestic and international studies on sulfuric acid hydrolysis of TBFS mainly focus on reaction processes and mechanisms (Ju et al., 2022), while there are fewer studies on the kinetic process of sulfuric acid hydrolysis. The following problems have arisen in actual process research: the acidolysis rate of each component in TBFS did not reach the equilibrium point of acidolysis kinetics, resulting in a high content of metal components in the remaining mud after acidolysis (Wang et al., 2022a). At the same time, the amount of mud increases and the component recovery decreases. This type of problem occurs not only in sulfuric acid hydrolysis of TBFS, but also in many sulfuric acid hydrometallurgy processes. Kinetic analysis can provide insight into the characteristics and mechanisms of sulphuric acid acidolysis of TBFS and predict the reaction rate, so as to effectively regulate the reaction conditions and improve the process efficiency. In order to provide a theoretical basis for the sulfuric acid acidolysis of TBFS, this study investigated the relationship between the total extraction rate of each component (i.e., all of Ti, Mg and Al) and the acidolysis time at different acidolysis temperatures, acid-slag ratios and raw material particle sizes. A kinetic model of the acidolysis process was fitted and the activation energy was calculated.

## 2 Experiment setup

### 2.1 Mineralogical analysis of TBFS

**FIGURE 1:**
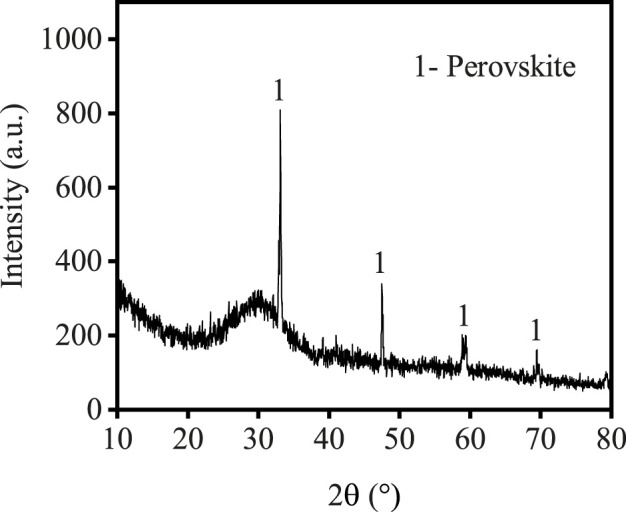
XRD patterns of TBFS.

The TBFS samples were dried at 106°C for 24 h and some TBFS samples were ground to a particle size of less than 0.074 mm for XRD and XRF testing. The XRD results in Figure 1 show that the main mineral phase is perovskite with some amorphous phase (Zhu et al., 2023; Li et al., 2024b). Table 1 shows the XRF analysis results of TBFS. The main chemical components are CaO, SiO2, TiO2, Al2O3 and MgO, with low contents of SO3, Fe2O3, K2O, MnO and Na2O, and the lowest contents of F, BaO, SrO and ZrO2.

**TABLE 1:** Chemical composition of TBFS.

Compound	wt%	Compound	wt%
CaO	28.08	K_2_O	0.72
SiO_2_	26.74	MnO	0.64
TiO_2_	19.65	Na_2_O	0.53
Al_2_O_3_	13.86	F	0.17
MgO	7.64	BaO	0.07
SO_3_	1.05	SrO	0.04
Fe_2_O_3_	0.79	ZrO_2_	0.02

**FIGURE 2:**
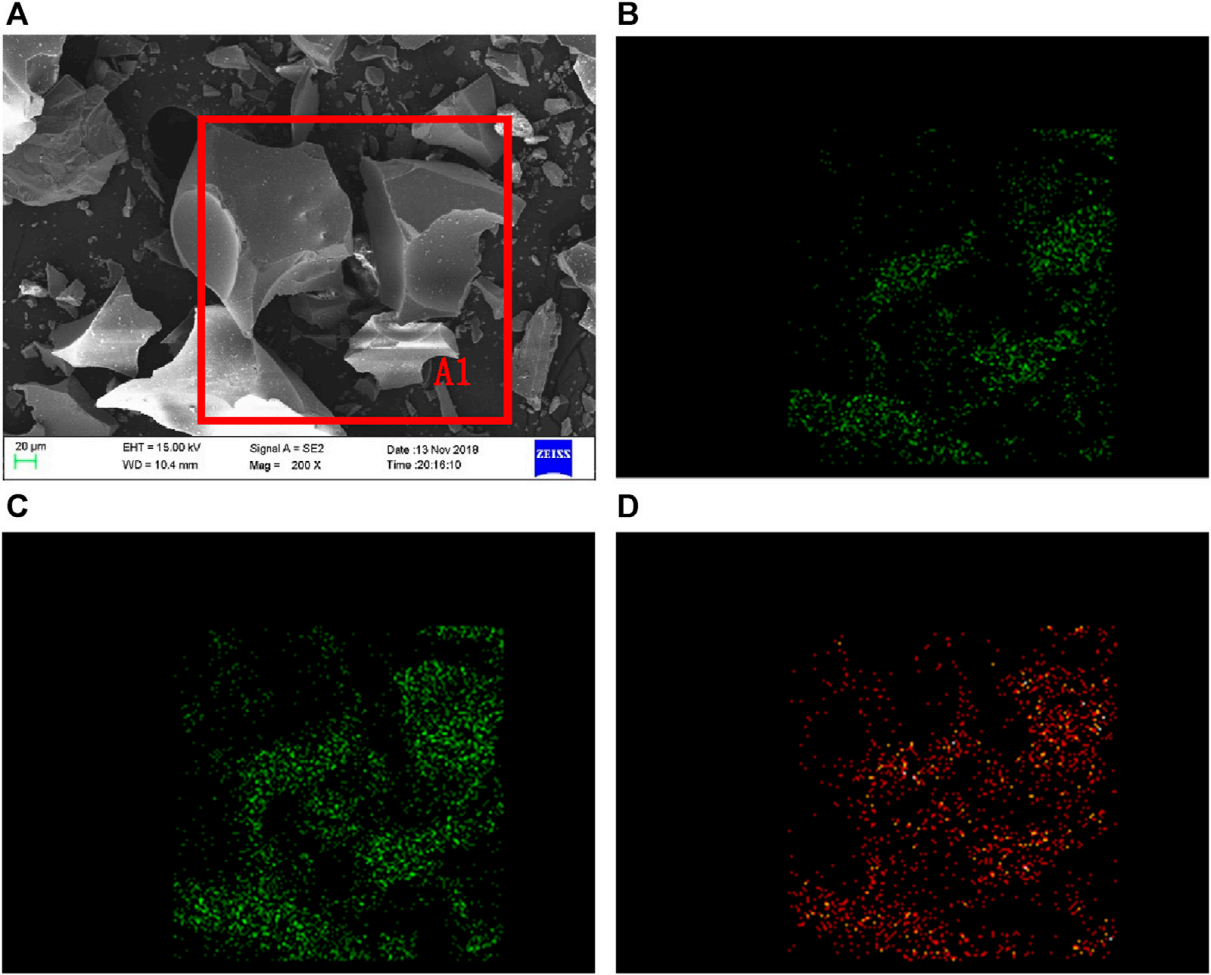
SEM-EDS spectrum of TBFS **(A)** SEM of TBFS **(B)** Mg element distribution **(C)** Al element distribution **(D)** Ti element distribution.

Figure 2 shows the SEM and EDS results of TBFS. From Figure 2A, it can be seen that the morphology of TBFS particles is an irregular structure with edges, smooth and dense surface, and uneven particle size. The EDS analysis was conducted on Ti, Mg, and Al elements in the A1 region. The distribution of each element is shown in Figures 2B–D. Ti, Mg, and Al elements are present on all visible particles and the enrichment zone boundary is not obvious, so the correlation between the three elements is well.

**FIGURE 3:**
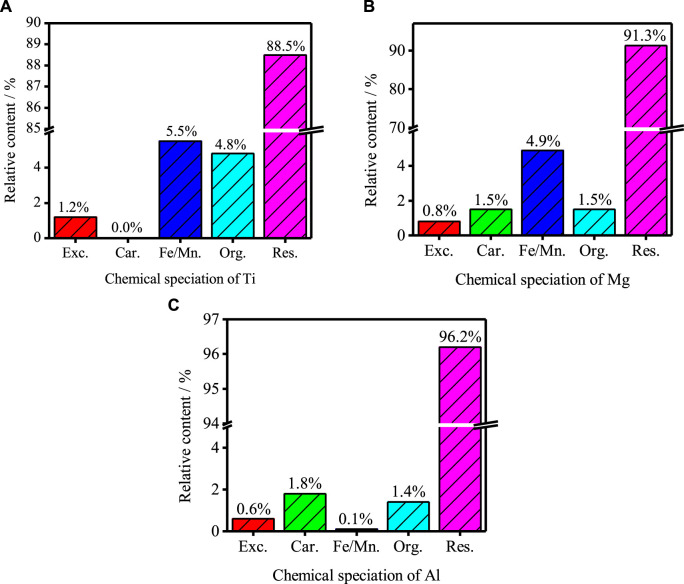
Occurrence state of Ti, Mg and Al in TBFS **(A)** Ti; **(B)** Mg; **(C)** Al.

Figure 3 shows the occurrence state of Ti, Mg and Al in TBFS determined by Tessier method. The percentage of Ti chemical form is residue state (58.5%) > oxidation state (35.5%) > organic state (4.8%) > exchangeable state (1.2%) > Ti carbonate state (0.0%). The percentage of Mg chemical form is residue state (91.3%) > oxidation state (4.9%) > organic state (1.5%) = carbonate state (1.5%) > exchangeable state (0.8%); The percentage of Al chemical form is residue state (96.2%) > carbonate state (1.8%) > organic state (1.4%) > exchangeable state (0.6%) > oxidation state (0.1%). The main occurrence of three components are residue state, indicating that their Chemical existence form is relatively stable.

### 2.2 Experimental procedure

a. Grind TBFS to the specific particle size shown in Table 2. Weigh the ground sample (10 g) in a 100 mL ceramic crucible with a lid and add concentrated sulfuric acid at the specific acid-slag mass ratio shown in Table 2.
b. After the acid and TBFS have been thoroughly mixed, the crucible is placed in a tubular program high-temperature furnace and the acidolysis reaction is carried out for a certain time at the specific temperature shown in Table 2. At the end of the reaction, the acidolysed slag was finely ground and 10 g of the slag is placed in a 150 mL conical flask, mixed with 60 mL of deionised water and placed in a water bath at 60°C with magnetic stirring at 20 rpm for 60 min.
c. After the water leaching, the leached slurry is vacuum filtered at 0.09 MPa to separate the liquid from the solid and separated into leach residue and leachate. The content of Ti, Mg and Al in the leachate is determined by chemical titration and the percentages of components extracted (R) were calculated according to Eq. 1 (Shangguan et al., 2022a; Ma et al., 2023):

$$R=\frac{m_{2}}{m_{1}}\times 100\%$$ (1)

**TABLE 2:** Design of kinetic experimental protocol.

Acidolysis temperature (°C)	Acid-slag ratio	TBFS particle size (μm)	Acidolysis time (min)
100, 110, 120, 130	1.4	Unclassified	5, 10, 15, 20, 40, 60
130	1.0, 1.2, 1.4, 1.6	Unclassified	5, 10, 15, 20, 40, 60
130	1.4	300, 200, 150, 74	5, 10, 15, 20, 40, 60

Here, m_1_ and m_2_ are the total masses of Ti, Al, and Mg components in the TBFS and leachate, respectively.

## 3 Results and discussion

It can be seen from Figure 1 that the main phase of TBFS is an amorphous structure, and the analysis in Figure 2 shows the high correlation between occurrence of Ti, Mg, and Al. Therefore, Ti, Mg, and Al are considered as a whole to calculate the total extraction rate of the components in the dynamic simulation (Nie et al., 2020; Nie et al., 2023b). The correlation calculation is carried out by investigating the relationship between the total extraction rate and the acidolysis time at different reaction temperatures, acid-slag ratio, and raw material particle sizes. The acidolysis reaction of concentrated sulphuric acid with TBFS is a typical liquid-solid reaction. Since the surface of the TBFS particles is relatively dense, it can be considered a non-porous structure. The particles containing Ti, Mg and Al gradually shrank during the reaction, so the most suitable model for the reaction kinetic is the unreacted shrinking core model (Alkan and Doğan, 2004; Zhang and Nicol, 2010; Liang et al., 2023a). The reaction started at the contact surface of concentrated H2SO4 and TBFS particles. The generated products (e.g., titanium sulphate) are wrapped around the surface of unreacted particles, and the H2SO4 diffused through the product layer to reach the unreacted interface to continue the acidolysis reaction. Thus, the overall rate of the reaction is affected by two steps: the internal diffusion step and the chemical reaction step (Sohn and Wadsworth, 2013; Wang et al., 2022b), the slower of which is the rate-controlling step during the acidolysis reaction process. Table 3 shows the relationship between the extraction rate r and time t for the two control steps of the unreacted shrinking core model.

**TABLE 3:** Integrated rate equation for unreacted shrinking core model.

Rate controlling step	Rate equation
Internal diffusion	1+2(1 – r) – 3(1 – r)^2/3^ = k_r_t
Chemical reaction	1 – (1 – r)^1/3^ = k_r_t

r: total extraction rate of Ti, Al or Mg; t: acidolysis time (min); kr: apparent rate constant.

**FIGURE 4:**
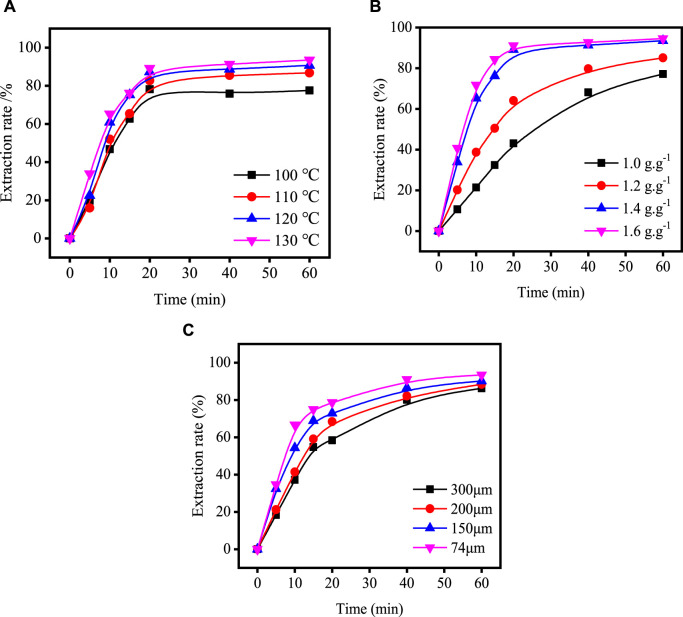
Variation in total extraction rate of components with acidolysis time under different process conditions: **(A)** acidolysis temperature; **(B)** acid-slag ratio; **(C)** TBFS particle size.

**FIGURE 5:**
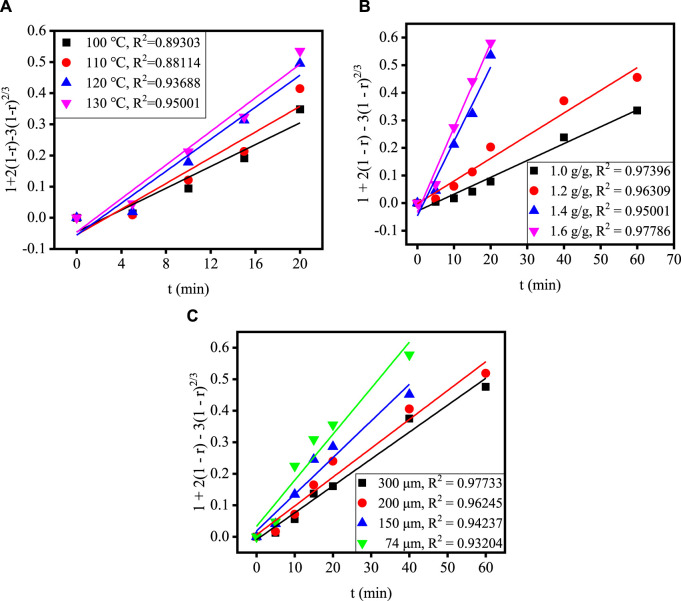
The relationship between 1 + 2(1 – r) – 3(1 – r)^2/3^ and t under different conditions: **(A)** acidolysis temperature, **(B)** acid-slag ratio and **(C)** TBFS particle size.

**FIGURE 6:**
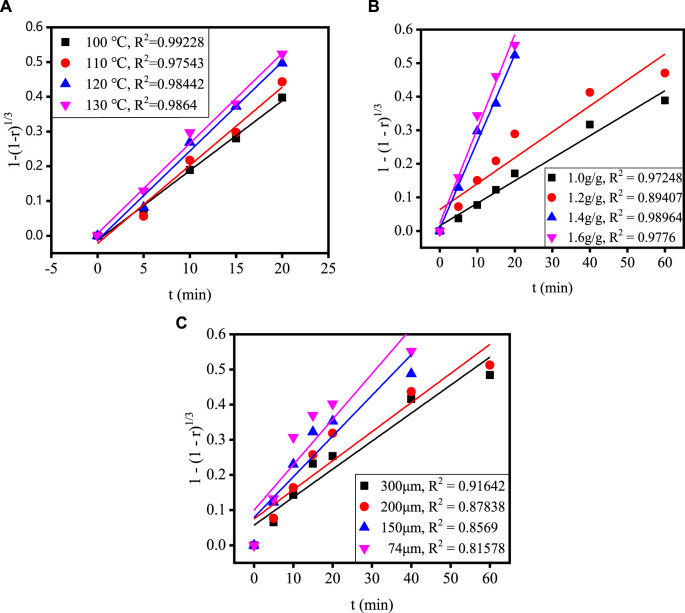
The relationship between 1 – (1 – r)^1/3^ and t under different conditions: **(A)** acidolysis temperature, **(B)** acid-slag ratio and **(C)** TBFS particle size.

Figure 4 shows the relationship between time and extraction rate at different acidolysis temperatures, acid-slag ratios and TBFS particle sizes. When the extraction rate tends to be stable, the acidolysis reaction is basically end, so only extraction rate before the end of acidolysis reaction can be used for calculation in the fitting calculation of the kinetic model. Therefore, the data before the reaction stabilizes in Figure 4 are fitted by substituting the equation in Table 3 and the fitting results are shown in Figures 5, 6. The kinetic fitting model is considered to be well fitted when the correlation coefficient (*R* ^2^) is greater than 0.85 (Wang et al., 2020; Li et al., 2023a), which shows that the *R* ^2^ between the internal diffusion Equation (1) + 2(1 – r) – 3(1 – r)^2/3^ and time t is greater than 0.85 for different acid-slag ratios, acidolysis temperatures and TBFS particle sizes. It can be assumed that the acidolysis reaction rate is mainly controlled by the internal diffusion of H_2_SO_4_ in the product layer (Zhang et al., 2023). The related fitting equation and reaction rate constant k_r_ are shown in Table 4.

**TABLE 4:** Fitting parameters of internal diffusion kinetic equations under different acidolysis conditions.

	Apparent rate ^constant^ k_r_	Fitting equation (t)
Acidolysis temperature (°C)		
100	0.01749	1 + 2(1 – r) – 3(1 – *r*)^2/3^ = 0.00661
110	0.02064	1 + 2(1 – r) – 3(1 – *r*)^2/3^ = 0.00914
120	0.02568	1 + 2(1 – r) – 3(1 – *r*)^2/3^ = 0.02568
130	0.02697	1 + 2(1 – r) – 3(1 – *r*)^2/3^ = 0.02985
Acid-slag ratio (g·g^–1^)		
1.0	0.00611	1 + 2(1 – r) – 3(1 – *r*)^2/3^ = 0.00611
1.2	0.00822	1 + 2(1 – r) – 3(1 – *r*)^2/3^ = 0.00822
1.4	0.02697	1 + 2(1 – r) – 3(1 – *r*)^2/3^ = 0.02697
1.6	0.03067	1 + 2(1 – r) – 3(1 – *r*)^2/3^ = 0.03067
TBFS particle size (μm)		
300	0.00854	1 + 2(1 – r) – 3(1 – *r*)^2/3^ = 0.00854
200	0.00916	1 + 2(1 – r) – 3(1 – *r*)^2/3^ = 0.00916
150	0.01163	1 + 2(1 – r) – 3(1 – *r*)^2/3^ = 0.02098
74	0.01461	1 + 2(1 – r) – 3(1 – *r*)^2/3^ = 0.01461

The apparent rate constant kr is influenced by the acidolysis temperature, the concentration of acidolysis solution in the reaction system and the feedstock radius, as shown in empirical Eq. 2 (Wang et al., 2013). Combined with the acidolysis conditions in this paper, this is translated into Eq. 3 to include the acid-slag ratio and TBFS particle size (Li et al., 2023b; Liang et al., 2023b).

$$k_{r}=\frac{kC_{2}M_{1}}{\rho_{1}r_{1}}$$ (2)

$$k_{r}=\frac{1.62xkM_{1}\rho_{2}}{r_{1}\left(1.62xM_{2}\rho_{1}+M_{2}\rho_{2}\right)}$$ (3)

Here, kr is the apparent rate constant, x is the acid-slag ratio, k is the temperature-dependent reaction rate constant, C2 is the concentration of concentrated sulphuric acid in the reaction system (the ratio of the molar concentration of sulphuric acid), M1 is the molar mass of TBFS,ρ2 is the density of the concentrated sulphuric acid, r1 is the radius of TBFS particles (mm); M2 is the molar mass of the concentrated sulphuric acid andρ1 is the density of TBFS.

The relationship between kr and the acidolysis temperature follows the Arrhenius Eq. 4. The logarithm of both sides of the equation is taken to obtain Eq. 5 (Shangguan et al., 2022b; Wang et al., 2022c).

$$k_{r}=Ae^{-E_{a}/RT}$$ (4)

$$\ln k_{r}=-\frac{E_{a}}{RT}+lnA$$ (5)

Here, kr is the apparent rate constant, A is the frequency factor, Ea is the apparent activation energy (J·mol–1), R is the molar gas constant (8.314 J mol–1) and T is the acidolysis temperature.

According to the data inTable 4, the relationship between lnkr and 1/T is plotted. As shown in Figure 6A, the apparent activation energy of the acidolysis reaction is estimated to be 19.05 kJ mol–1 based on the slope of the straight line in Eqss 4, and A was estimated as 8.23 based on the intercept. It is generally accepted that higher activation energies (>40 kJ mol^–1^) indicate chemical control, while activation energies of <20 kJ mol^–1^ indicate diffusion-controlled processes (Habashi, 1980; Santos et al., 2010). Thus, these results further indicated that the acidolysis process is consistent with internal diffusion control. The relationship between k r and the acidolysis temperature T could be expressed as Eq. 6.

$$k_{r}=8.23e^{-19050/RT}$$ (6)

When $\frac{r_{1}M_{2}}{kM_{1}}$ is replaced by constant A1, Eq. 3 is transformed into an equation relating kr to the acid-slag ratio x by taking the reciprocal and then the logarithm of both sides, as shown in Eq. 7 ^.^

$$\ln k_{r}^{-1}=\ln A_{1}+\ln\left(\frac{\rho_{1}}{\rho_{2}}+\frac{1}{1.62x}\right)$$ (7)

The relationship between lnk_r_ and $\ln\left(\frac{\rho_{1}}{\rho_{2}}+\frac{1}{1.62x}\right)$ is shown in Figure 6B. The slope of the fitted line is 9.80201, so the relationship between k_r_ and x can be expressed as Eq. 8.

$$k_{r}^{-1}=A_{1}{\left(\frac{\rho_{1}}{\rho_{2}}+\frac{1}{1.62x}\right)}^{9.80201}$$ (8)

When $\frac{1.62xkM_{1}\rho_{2}}{\left(1.62xM_{2}\rho_{1}+M_{2}\rho_{2}\right)}$ is replaced by the constant A_2_, Eq. 3 can be converted into Eq. 9.

$$\ln k_{r}=\ln A_{2}-\ln r_{1}$$ (9)

**FIGURE 7:**
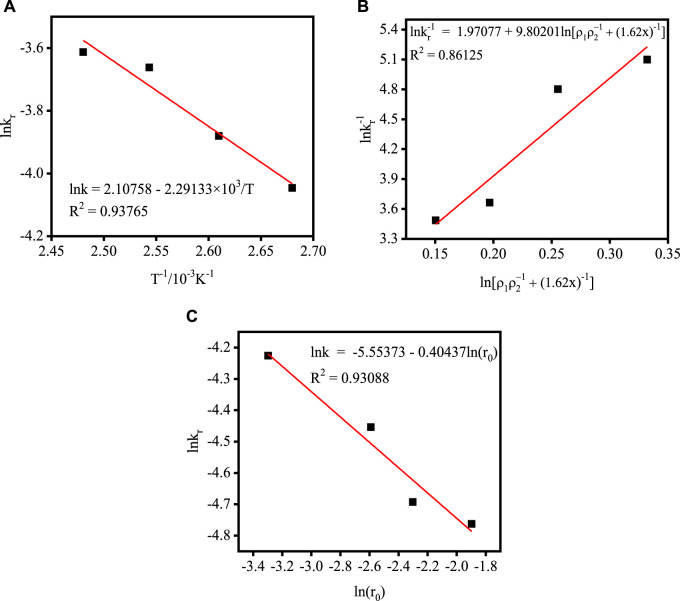
Plot of k_r_ *versus* different process parameters: **(A)** acidolysis temperature; **(B)** acid-slag ratio; **(C)** TBFS particle radius.

Figure 7C shows the relationship between lnk_r_ and lnr_1_. The slope of the fitted line is −0.40437, so the relationship between k_r_ and r_1_ can be expressed as Eq. 10.

$$k_{r}=\frac{A_{2}}{r_{1}^{0.40437}}$$ (10)

Combining Eqs 6, 8, 10, the semi-empirical kinetic equations related to the acid-slag ratio, acidolysis temperature, TBFS particle radius and k_r_ are established as follows.

$$k_{r}=\frac{kM_{1}}{r_{1}M_{2}}{\left(\frac{\rho_{1}}{\rho_{2}}+\frac{1}{1.62x}\right)}^{-1}=\frac{kM_{1}}{r_{1}^{0.40437}M_{2}}{\left(\frac{\rho_{1}}{\rho_{2}}+\frac{1}{1.62x}\right)}^{-9.80201}$$

$$=\frac{M_{1}}{r_{1}^{0.40437}M_{2}}{\left(\frac{\rho_{1}}{\rho_{2}}+\frac{1}{1.62x}\right)}^{-9.80201}A_{3}e^{-E_{a}/RT}$$

$$=\frac{M_{1}}{r_{1}^{0.40437}M_{2}}{\left(\frac{\rho_{1}}{\rho_{2}}+\frac{1}{1.62x}\right)}^{-9.80201}A_{3}e^{-19050/RT}$$

$$=\frac{{\left(\frac{\rho_{1}}{\rho_{2}}+\frac{1}{1.62x}\right)}^{-9.80201}}{r_{1}^{0.40437}}A^{\prime }e^{-19050/RT}$$

Here, A_3_ is the frequency factor and $A^{\prime }=\frac{M_{1}A_{3}}{M_{2}}$ .

Since $k_{r}=8.23e^{-19050/RT}$ , so $\frac{{\left(\frac{\rho_{1}}{\rho_{2}}+\frac{1}{1.62x}\right)}^{-9.80201}}{r_{1}^{0.40437}}A^{\prime }=8.2$ . Substituting ρ_1_ = 1.429, ρ_2_ = 1.84 and the process parameters with the best fit to the internal diffusion control equation; that is, x = 1.6 and r_1_ = 75 × 10^−6^ m, it is calculated that $A^{\prime }$ = 77.29 × 10^−2^. Therefore, the kinetic equation could be shown as Eq. 11.

$$1+2\times \left(1-r\right)-3\times {\left(1-r\right)}^{\frac{2}{3}}=\frac{{1.02\times \left(0.78+\frac{1}{1.62x}\right)}^{-9.80201}}{d_{1}^{0.40437}}e^{-19050/RT}\times t$$ (11)

Here, x is the acid-slag ratio, d_1_ is the TBFS particle size (m), R is the molar gas constant (R = 8.314 J mol^–1^) and T is the acidolysis temperature (K).

**FIGURE 8:**
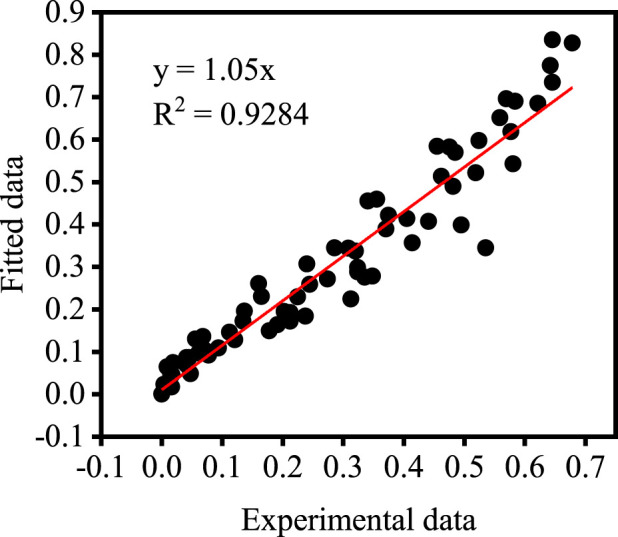
Comparison of experimental results and fitted results.

The data in Figure 4 are substituted into the left-hand side of Eq. 11 and the corresponding values of the relevant process parameters are substituted into the right-hand side of Eq. 11 for comparative analysis. Figure 8 shows the results of the comparison between experimental and fitted values, which are in good correlation with each other. This indicates that the derived kinetic equations are valuable in reflecting the actual situation.

**FIGURE 9:**
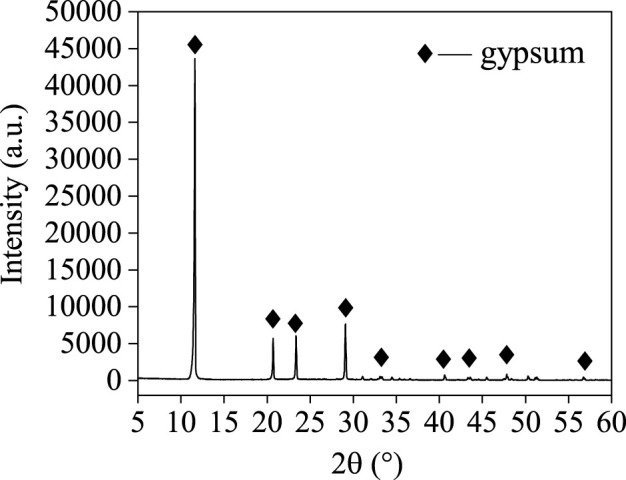
XRD patterns of water leached residues.

Under the conditions of average particle size of 150 μm, acid/solid ratio of 1.4, acidolysis temperature of 300°C, and acidolysisi time of 40 min, the extraction rates of Ti, Mg, and Al reached 82.85%, 93.16%, and 96.96%, respectively. The chemical analysis and XRD of the leached residue are shown in Table 5 and Figure 9. After acidolysis with sulfuric acid and leaching with deionized water, the main elements in residual are S, Si, and Ca, with Ca mainly present in the form of gypsum.

**TABLE 5:** Chemical composition of the leaching resides with titanium leaching rate at 82.5%.

Compound	wt%	Compound	wt%
SO_3_	36.84	K_2_O	0.12
SiO_2_	31.09	MnO	0.09
CaO	24.38	Na_2_O	0.07
TiO_2_	5.26	BaO	0.05
Al_2_O_3_	1.24	SrO	0.03
MgO	0.58	ZrO_2_	0.01
Fe_2_O_3_	0.25		

## 4 Conclusion

a. The chemical composition of TBFS is complex, with the main chemical componets are CaO, SiO_2_, TiO_2_, Al_2_O_3_, and MgO. There is a certain correlation and close connection between different elements in TBFS, and the main mineral phase of TBFS is perovskite, and there are also some amorphous structures.
b. The acidolysis temperature, acid-slag ratio and TBFS particle size are positively correlated with the extraction rate of TBFS, with the higher the value of these three variables, the higher the extraction rate.
c. The acidolysis process is consistent with the ‘unreacted shrinking core model’ and the reaction rate is controlled by internal diffusion through the solid product layer.
d. The apparent activation energy of the acidolysis reaction is calculated by the Arrhenius equation as 19.05 kJ mol^–1^ and the kinetic equation is:

$$1+2\times \left(1-r\right)-3\times {\left(1-r\right)}^{\frac{2}{3}}=\frac{{1.02\times \left(0.78+\frac{1}{1.62x}\right)}^{-9.80201}}{d_{1}^{0.40437}}e^{-19050/RT}$$

## Data Availability

The original contributions presented in the study are included in the article/Supplementary Material, further inquiries can be directed to the corresponding authors.

## References

[B1] Alkan M. Doğan M. (2004). Dissolution kinetics of colemanite in oxalic acid solutions. Chem. Eng. Process. Process Intensif. 43 (7), 867–872. 10.1016/s0255-2701(03)00108-9

[B2] Habashi F. (1980). Principles of extractive metallurgy. General Principles, 1. New York: Gordon and Breach.

[B3] He S. Peng T. Sun H. (2019). Titanium recovery from Ti-bearing blast furnace slag by alkali calcination and acidolysis. Jom 71 (9), 3196–3201. 10.1007/s11837-019-03575-9

[B4] He S. Wang Y. (2023). Extraction of valuable components from Ti-bearing blast furnace slag using sulfuric acid calcination process. JOM 75 (2), 392–399. 10.1007/s11837-022-05557-w

[B5] Hongjuan S. Guobiao Z. Tongjiang P. Xiao W. Siqi H. Fan Z. (2015). Recovery of titanium from titanium-rich product prepared from high ti-bearing blast furnace slag by sulfuric acid leaching. Min. metallurgy 24 (3), 54–58.

[B6] Huang S. Zhang R. (1994). Pilot test of carbonization of the molten blast furnace TiO2 slag at PanZhiHua iron and steel company. Iron Steel Vanadium Titan. 15 (2), 17–21.

[B7] Huang X. Wang Z. Liu Y. Hu W. Ni W. (2016). On the use of blast furnace slag and steel slag in the preparation of green artificial reef concrete. Constr. Build. Mater. 112, 241–246. 10.1016/j.conbuildmat.2016.02.088

[B8] Jiang T. Dong H. Guo Y. Li G. Yang Y. (2010). Study on leaching Ti from Ti bearing blast furnace slag by sulphuric acid. Mineral Process. Extr. Metallurgy 119 (1), 33–38. 10.1179/037195509x12585446038807

[B9] Ju J. Feng Y. Li H. Wu R. Wang B. (2022). An approach towards utilization of water-quenched blast furnace slag for recovery of titanium, magnesium, and aluminum. J. Environ. Chem. Eng. 10 (4), 108153. 10.1016/j.jece.2022.108153

[B10] Kuwahara Y. Ohmichi T. Kamegawa T. Mori K. Yamashita H. (2010). A novel conversion process for waste slag: synthesis of a hydrotalcite-like compound and zeolite from blast furnace slag and evaluation of adsorption capacities. J. Mater. Chem. 20 (24), 5052–5062. 10.1039/c0jm00518e

[B11] Li W. X. Liu M. S. Cheng S. B. Zhang H. F. Yang W. X. Yi Z. (2024a). Polarization independent tunable bandwidth absorber based on single-layer graphene. Diam. Relat. Mater. 142, 110793. 10.1016/j.diamond.2024.110793

[B12] Li W. X. Ma J. Zhang H. F. Cheng S. B. Yang W. X. Yi Z. (2023c). Tunable broadband absorber based on a layered resonant structure with a Dirac semimetal. Phys. Chem. Chem. Phys. 25, 8489–8496. 10.1039/d2cp05562g 36883439

[B13] Li W. X. Xu F. Cheng S. B. Yang W. X. Liu B. Liu M. S. (2024b). Six-band rotationally symmetric tunable absorption film based on AlCuFe quasicrystals. Opt. Laser Technol. 169, 110186. 10.1016/j.optlastec.2023.110186

[B14] Li W. X. Yi Y. T. Yang H. Cheng S. B. Yang W. X. Zhang H. F. (2023b). Active tunable terahertz bandwidth absorber based on single layer graphene. Commun. Theor. Phys. 75, 045503. 10.1088/1572-9494/acbe2d

[B15] Li W. X. Zhao W. C. Cheng S. B. Yang W. X. Yi Z. Li G. F. (2023a). Terahertz selective active electromagnetic absorption film based on single-layer graphene. Surfaces Interfaces 40, 103042. 10.1016/j.surfin.2023.103042

[B16] Liang S. R. Xu F. Li W. X. Yang W. X. Cheng S. B. Yang H. (2023a). Tunable smart mid infrared thermal control emitter based on phase change material VO2 thin film. Appl. Therm. Eng. 232, 121074. 10.1016/j.applthermaleng.2023.121074

[B17] Liang S. R. Xu F. Yang H. Cheng S. B. Yang W. X. Yi Z. (2023b). Ultra long infrared metamaterial absorber with high absorption and broad band based on nano cross surrounding. Opt. Laser Technol. 158, 108789. 10.1016/j.optlastec.2022.108789

[B18] Ma J. Wu P. H. Li W. X. Liang S. R. Shangguan Q. Y. Cheng S. B. (2023). A five-peaks graphene absorber with multiple adjustable and high sensitivity in the far infrared band. Diam. Relat. Mater. 136, 109960. 10.1016/j.diamond.2023.109960

[B19] Nie W. Wen S. Feng Q. Liu D. Zhou Y. (2020). Mechanism and kinetics study of sulfuric acid leaching of titanium from titanium-bearing electric furnace slag. J. Mater. Res. Technol. 9 (2), 1750–1758. 10.1016/j.jmrt.2019.12.006

[B20] Nie W. Wen S. Liu D. Hu T. Zhang L. (2023a). Innovative application of two-stage sulfuric acid leaching for efficient recovery of Ti from titanium-bearing electric furnace slag. J. Environ. Chem. Eng. 11 (1), 109174. 10.1016/j.jece.2022.109174

[B21] Nie W. Wen S. Liu D. Hu T. Zhang L. (2023b). Innovative application of two-stage sulfuric acid leaching for efficient recovery of Ti from titanium-bearing electric furnace slag. J. Environ. Chem. Eng. 11 (1), 109174. 10.1016/j.jece.2022.109174

[B22] Peng Y. Ao J. Xia Q. (2005). The causes and countermeasures for non-hydrated activity of residual slags from chlorination process of PanGang BF slags. Multipurp. Util. Mineral Resour. 6, 40–46.

[B23] Santos F. M. Pina P. S. Porcaro R. Oliveira V. A. Silva C. A. Leão V. A. (2010). The kinetics of zinc silicate leaching in sodium hydroxide. Hydrometallurgy 102 (1-4), 43–49. 10.1016/j.hydromet.2010.01.010

[B24] Shangguan Q. Y. Chen H. Yang H. Liang S. R. Zhang Y. J. Cheng S. B. (2022b). A “belfry-typed” narrow-band tunable perfect absorber based on graphene and the application potential research. Diam. Relat. Mater. 125, 108973. 10.1016/j.diamond.2022.108973

[B25] Shangguan Q. Y. Zhao Y. Song Z. J. Wang J. Yang H. Chen J. (2022a). High sensitivity active adjustable graphene absorber for refractive index sensing applications. Diam. Relat. Mater. 128, 109273. 10.1016/j.diamond.2022.109273

[B26] Sohn H. Y. Wadsworth M. E. (2013). Rate processes of extractive metallurgy. Germany: Springer Science and Business Media.

[B27] Valighazvini F. Rashchi F. Nekouei R. K. (2013). Recovery of titanium from blast furnace slag. Industrial Eng. Chem. Res. 52 (4), 1723–1730. 10.1021/ie301837m

[B28] Wang D. Y. Yi Z. Ma G. L. Dai B. Yang J. B. Zhang J. F. (2022c). Two-channel photonic crystal fiber based on surface plasmon resonance for magnetic field and temperature dual-parameter sensing. Phys. Chem. Chem. Phys. 24, 21233–21241. 10.1039/d2cp02778j 36040374

[B29] Wang D. Y. Zhu W. L. Yi Z. Ma G. L. Gao X. Dai B. (2022b). Highly sensitive sensing of a magnetic field and temperature based on two open ring channels SPR-PCF. Opt. Express 30, 39055. 10.1364/oe.470386 36258455

[B30] Wang L. Chen L. Liu W. Zhang G. Tang S. Yue H. (2022a). Recovery of titanium, aluminum, magnesium and separating silicon from titanium-bearing blast furnace slag by sulfuric acid curing—leaching. Int. J. Minerals, Metallurgy Mater. 29 (9), 1705–1714. 10.1007/s12613-021-2293-3

[B31] Wang M. Z. Chen J. Jing B. Y. Zhang L. Y. Dong Y. Y. Yu X. Z. (2020). Analysis of reaction kinetics of edible oil oxidation at ambient temperature by FTIR spectroscopy. Eur. J. Lipid Sci. Technol. 122, 1900302. 10.1002/ejlt.201900302

[B32] Wang W. Zeng D. Chen Q. Yin X. (2013). Experimental determination and modeling of gypsum and insoluble anhydrite solubility in the system CaSO4–H2SO4–H2O. Chem. Eng. Sci. 101, 120–129. 10.1016/j.ces.2013.06.023

[B33] Zhang C. Yi Y. T. Yang H. Yi Z. Chen X. F. Zhou Z. G. (2022). Wide spectrum solar energy absorption based on germanium plated ZnO nanorod arrays: energy band regulation, Finite element simulation, Super hydrophilicity, Photothermal conversion. Appl. Mater. Today 28, 101531. 10.1016/j.apmt.2022.101531

[B34] Zhang L. Zhang L. Wang M. Li G. Sui Z. (2007). Recovery of titanium compounds from molten Ti-bearing blast furnace slag under the dynamic oxidation condition. Miner. Eng. 20 (7), 684–693. 10.1016/j.mineng.2007.01.003

[B35] Zhang S. Nicol M. J. (2010). Kinetics of the dissolution of ilmenite in sulfuric acid solutions under reducing conditions. Hydrometallurgy 103 (1-4), 196–204. 10.1016/j.hydromet.2010.03.019

[B36] Zhang Y. J. Yi Y. T. Li W. X. Liang S. R. Ma J. Cheng S. B. (2023). High absorptivity and ultra-wideband solar absorber based on Ti-Al2O3 cross elliptical disk arrays. Coatings 13 (3), 531. 10.3390/coatings13030531

[B37] Zhou G. Peng T. Sun H. Liang Y. (2013). The products transformation and formation mechanism in the roasting process of high Ti-bearing blast furnace slag with ammonium sulfate. Acta Petrologica Mineralogica 32 (6), 893–898.

[B38] Zhou Z. Zhang B. Zhu Z. (1999). A test of titania separation from high titania bearing blast furnace slag. Iron Steel Vanadium Titan. 20 (4), 37–40.

[B39] Zhu W. L. Yi Y. T. Yi Z. Bian L. Yang H. Zhang J. G. (2023). High confidence plasmonic sensor based on photonic crystal fibers with a U-shaped detection channel. Phys. Chem. Chem. Phys. 25, 8583–8591. 10.1039/d2cp04605a 36883940

